# First-principles study of the surface reactions of aminosilane precursors over WO_3_(001) during atomic layer deposition of SiO_2_†

**DOI:** 10.1039/d0ra01635g

**Published:** 2020-04-27

**Authors:** Kyungtae Lee, Youngseon Shim

**Affiliations:** CSE Team, Data & Information Technology (DIT) Center, Samsung Electronics Co. Ltd. 130 Samsung-ro Suwon Gyeonggi-do 16678 South Korea ys1231.shim@samsung.com

## Abstract

Atomic layer deposition (ALD) has emerged as a critical technique to deposit highly conformal and uniform thin films for advanced semiconductor devices. The development of ALD processes relies on ALD precursor design to meet the required properties of thin films. In this study, we report the ALD mechanisms of silicon oxide over the tungsten oxide substrate using density functional theory (DFT) methods. To analyze the ligand effects of precursors, we compare the surface reactions of different aminosilane precursors with a varying number of amino ligands such as diisopropylaminosilane (DIPAS), bis(diethylamino)silane (BDEAS), and tris(dimethylamino)silane (TDMAS) over the hydroxyl-terminated WO_3_ (001) surface. BDEAS shows the lowest energy barrier in the rate-determining step and the overall reaction energetics of DIPAS and BDEAS decomposition are more exothermic than that of TDMAS. For this reason, BDEAS is estimated to provide the fastest growth rate. However, the binding strength of the leaving amine molecule of DIPAS on WO_3_(001) is weaker than those of BDEAS and TDMAS, and thus DIPAS is more likely to reduce surface impurities during the ALD process.

## Abstract 2

Energy diagram of reaction pathways for decomposition of different aminosilane precursors on a WO_3_ (001) surface.

## Graphical abstract

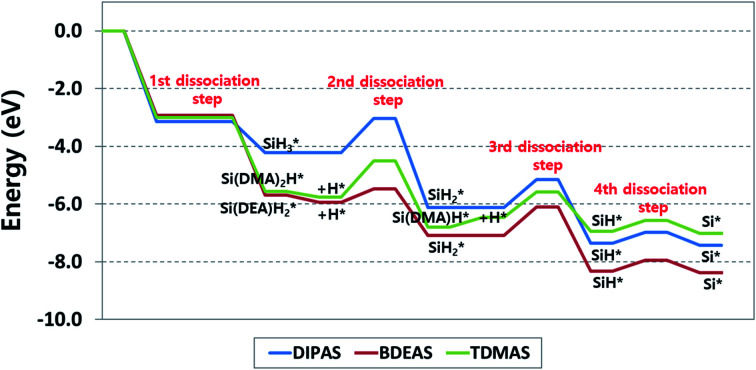

## 1. Introduction

The semiconductor industry has continued efforts of miniaturizing memory devices to obtain a competitive edge. This technology trend results in more integrated semiconductor devices with three-dimensional structures and high aspect ratio, generating a demand for highly uniform and conformal thin films.^1–3^ Among thin-film deposition techniques, atomic layer deposition (ALD) allows for atomic level control of the thin film deposition by self-limiting surface reactions, enabling excellent step coverage at low temperatures.^4,5^ During the ALD process, the precursors are added to the ALD reactor and all the reactive sites on the substrate are consumed. The remaining precursors are purged out by inert gas or vacuum. This reaction cycle leads to the limiting growth rate, and thus ALD is widely used for a high quality of thin film deposition over various shapes of substrates at a nanometer scale.^6^

Silicon dioxide (SiO_2_) has been used as a representative dielectric material in the microelectronics industry, due to its good dielectric and etching properties.^7,8^ There are various types of ALD precursors to grow SiO_2_ thin film such as chlorosilanes,^9,10^ alkoxides,^11^ and alkylamides.^12,13^ Generally, precursors are required to show good volatility for the gas-phase process and also good thermal stability in gas-phase in order to reach the surface without decomposition. Once precursors adsorb on surfaces, they should be reactive enough to consume all the reactive sites before the next purge of inert gas.^14,15^ Halide-based precursors are well utilized for ALD of silicon nitride with nitrogen agents such as NH_3_ or N_2_, however, show severe limitations for ALD of silicon oxide due to high temperature conditions required and chlorine impurities in the film.^16^ Aminosilane precursors containing alkylamides are chlorine-free and highly reactive, enabling a fast growth rate of a high quality of SiO_2_ thin film at low temperatures.^17,18^

Aminosilane precursors dissociatively adsorb as silane and amino fragments through the Si–N bond breaking catalyzed by a surface OH group.^19,20^ While the silane fragment is attached to a surface oxygen atom and take a part in the next dissociation reaction, the amino fragment is removed as a gas molecule at the ALD purge step. A different number of amino groups along with structural variations affects the ALD performance of aminosilane precursors. A previous experimental study showed that the ALD growth rate and film purity were improved by using bis-dimethylaminosilane (BDMAS) with two alkylamino ligands as compared to tris-dimethylaminosilane (TDMAS) with three alkylamino ligands.^21^

Computational studies have been also conducted to study the effects of ligand size and number of aminosilane precursors on the ALD reactions. The decomposition reactions of Si precursors containing two alkylamino ligands are thermodynamically and kinetically favorable on OH-terminated Si or SiO_2_ surfaces,^19,22,23^ whereas the ALD with TDMAS is energetically unfavorable,^24^ consistent with the previous experimental result.^21^ The final deligation step of TDMAS was found to be an endothermic reaction with a high activation energy of more than 70 kcal mol^−1^, limiting the overall ALD performance of TDMAS. Another computational study reported that both BDMAS and TDMAS showed relatively low reaction barriers and acceptable adsorption energies in the first dissociation step.^17^ Mono-alkylamino silane precursors with different ligand sizes were also analyzed by DFT calculations where DIPAS exhibited the widest ALD window among six precursors.^25^ However, several Si precursors have not been analyzed systematically on a surface slab using computational methods over the entire reaction paths of precursor decomposition up to the Si seed formation, so there is still a lack of understanding of overall reaction energetics across precursors.

We previously reported the full reaction energetics of DIPAS on tungsten surfaces as one of gate metals used in complementary metal-oxide-semiconductor (CMOS) devices.^26^ In particular, we examined the OH-terminated tungsten oxide surface, as tungsten is easily oxidized during the ALD processes. Even if the substrate is different from a silicon oxide surface, the reaction pathway of DIPAS decomposition on the WO_3_ (001) surface was found to be comparable to that on the SiO_2_ surfaces due to the structural similarity involving OH-terminated surfaces. Herein, we report the full reaction energetics of three different Si precursors from mono to tri alkylamino ligands, such as DIPAS, BDEAS, and TDMAS on the WO_3_ (001) surface. To the best of our knowledge, this is the first study to investigate the full reaction energetics of multiple Si precursors on a tungsten oxide surface. The key issue we undertake in this article is how Si ALD processes are influenced by different substrates and precursors. To address this issue, we examine the impact of the number of amino functional groups on precursor adsorption and decomposition by comparing the calculated reaction energetics of three precursors.

## 2. Methods

### 2.1. Computational methods

Density functional theory (DFT) calculations were performed using the Vienna *Ab initio* Simulation Program (VASP), an *ab initio* total energy and molecular dynamics program developed at the University of Vienna.^27,28^ The electron–ion interactions were considered by the projector augmented wave (PAW) description of atomic cores,^29,30^ where the electron exchange–correlation energies were described by the Perdew–Burke–Ernzerhof (PBE) functional^31^ and the dispersion corrections were made by the D3 method.^32^ The electronic wave functions were represented by a plane wave basis set with a cutoff energy of 400 eV. The surface Brillouin zone was sampled with 2 × 2 × 1 *k*-point based on the gamma centered grid for WO_3_. All geometry optimizations were performed for the forces of all atoms to be less than 0.05 eV Å^−1^ except for the WO_3_ crystal structure which was optimized to be less than 1 × 10^−6^ eV Å^−1^. To verify the effect of convergence criteria of forces, the surface slab energy was tested with the force convergence criteria of 0.01 and 0.03 eV Å^−1^ in which the differences compared to the result of 0.05 eV Å^−1^ were calculated to be within 0.03 eV. We also calculated the reaction energetics of the first Si–N dissociation of DIPAS on the hydroxyl-terminated WO_3_ (001) using a force convergence criterion of 0.03 eV Å^−1^ as shown in Fig. S1 (ESI†). There was no change in the reaction barrier with the reaction energy lowered only by 0.02 eV compared to the result of 0.05 eV Å^−1^, suggesting that it is reasonable to use the criterion of 0.05 eV Å^−1^.

**Fig. 1:**
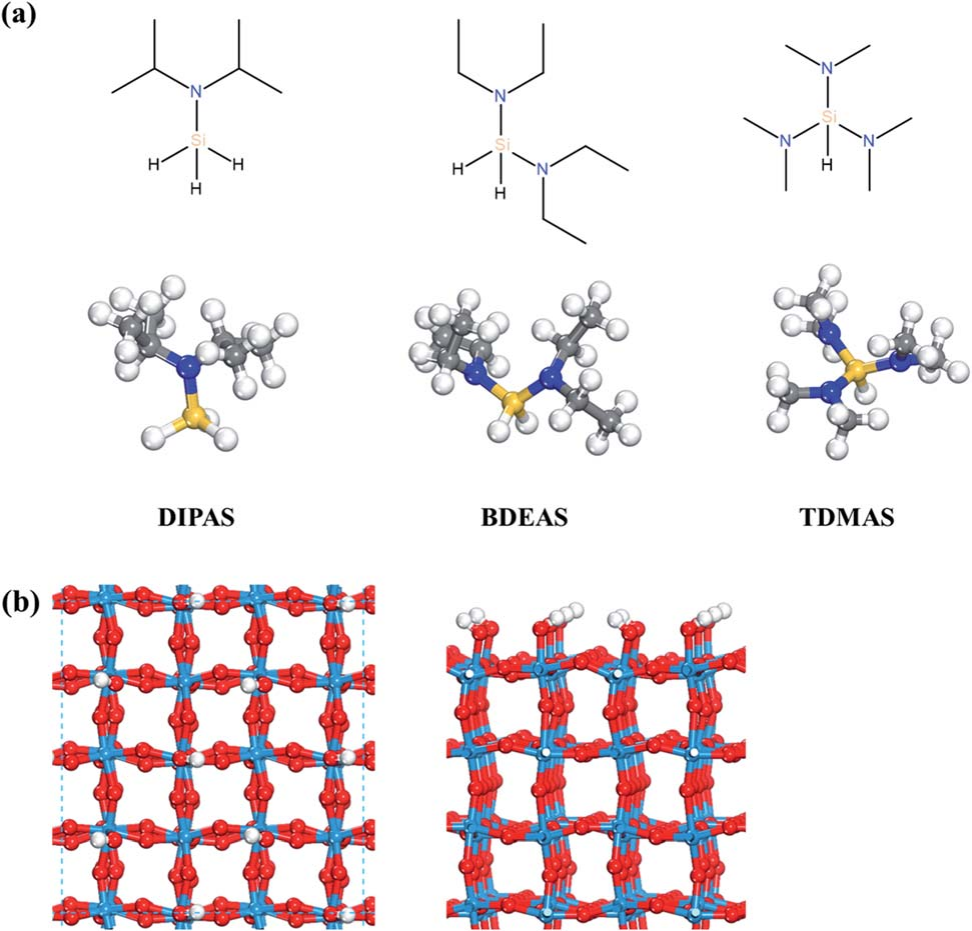
(a) Structures of three Si precursors and (b) top and side views of the hydroxyl-terminated WO_3_ (001) surface. Red spheres, O; light blue spheres, W; blue spheres, N; yellow spheres, Si; gray spheres, C; white spheres, H.

All isolated molecules were optimized in a 20 × 20 × 20 Å unit cell. The Si precursors considered in this study such as DIPAS [SiH_3_NR_2_, R = CH(CH_3_)_2_], BDEAS [SiH_2_(NR_2_)_2_, R = CH_2_CH_3_], and TDMAS [SiH(NR_2_)_3_, R = CH_3_] are shown in Fig. 1a. Native tungsten oxide surfaces are modeled in this study by a crystalline surface slab, as described in our previous report.^26^ ALD reactions were simulated on the WO_3_ (001) surface, as the monoclinic (001) surface is the most favorable at room temperature for WO_3_.^33^ The lattice parameters of the bulk monoclinic WO_3_ after structural optimization were 7.300 Å, 7.530 Å, 7.680 Å, and 90.9° for *a*, *b*, *c*, and *β*, respectively, consistent with the experiment.^34,35^ The WO_3_ (001) surface slab was prepared based on previous experimental and computational studies.^36–40^ A 4 × 4 unit cell was used for the surface slab with four atomic layers where top two layers were relaxed (Fig. 1b). The terminal oxygen atoms on WO_3_ (001) surface were altered to hydroxyl groups, so as to model the preferred adsorption of atomic hydrogen on the terminal oxygen atoms.^41^ The surface coverage effect associated with the cell size of the surface slab was examined by comparing the adsorption energies of DIPAS on two different cell sizes of WO_3_ surfaces such as the 4 × 4 and 6 × 6 unit cells as shown in Fig. S2 (ESI†). The energy difference is calculated to be 0.05 eV, suggesting that the use of the 4 × 4 cell unit allows for computing the reaction energetics of three Si precursors with minimizing the lateral interactions of adsorbates across the periodic surface slabs.

**Fig. 2:**
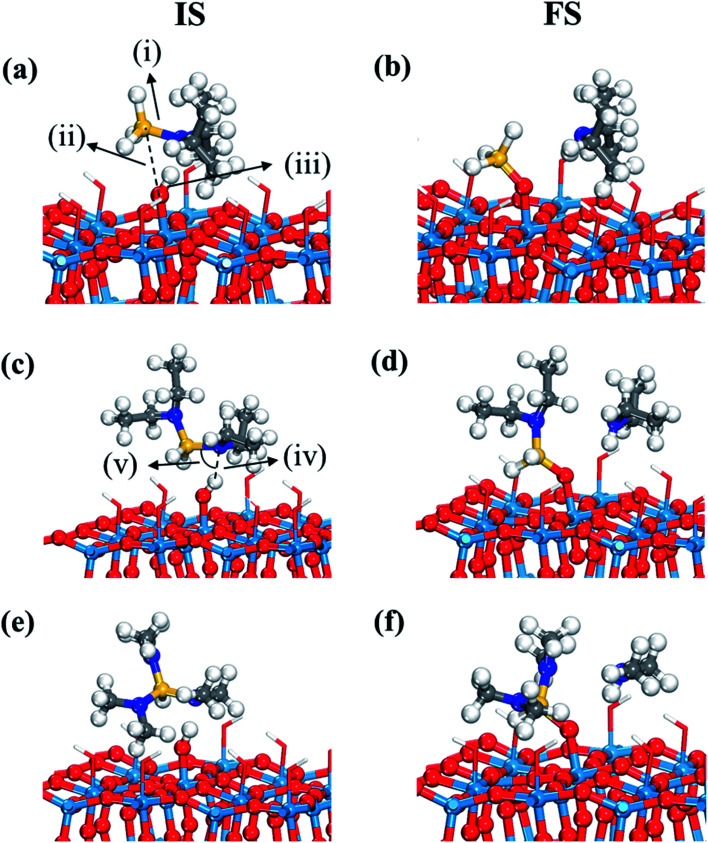
Initial (IS) and final (FS) states of the first dissociation step, dissociative chemisorption, of three precursors such as (a and b) DIPAS, (c and d) BDEAS, (e and f) TDMAS. Terminal hydroxyl groups on the WO_3_ surface are displayed in stick style if there is no direct interaction with adsorbates, in which red and white colors indicate O and H atoms, respectively. Red spheres, O; light blue spheres, W; blue spheres, N; yellow spheres, Si; gray spheres, C; white spheres, H. (i) Si–N distance (1.771 (a); 3.605 (b); 1.772 (c); 4.041 (d); 1.783 (e); 3.987 (f)), (ii) Si–O distance (3.060 (a); 1.648 (b); 2.686 (c); 1.644 (d); 2.969 (e); 1.639 (f)), (iii) O–H distance (1.024 (a); 2.608 (b); 1.002 (c); 2.722 (d); 1.034 (e); 2.496 (f)), (iv) N–H distance (1.666 (a); 1.024 (b); 1.819 (c); 1.023 (d); 1.592 (e); 1.024 (f)), and (v) H–N–Si angle (88.4° (a); 82.3° (c); 87.9° (e)). The unit of distance is Å.

For transition state search, the climbing-image nudged elastic band (CI-NEB) method was used.^42,43^ Each transition state was isolated by interpolating and optimizing four equally spaced images between the initial and final states, confirmed by an absolute tangential force below 0.05 eV Å^−1^ and an imaginary vibration frequency along with an analysis of relevant vibration dynamics. In the difficult cases of identifying a transition state, we narrowed down the searching region by selecting new initial and final states among four images, accordingly more than four images were used to find a transition state. For example, the activation energy and reaction energy of dissociative chemisorption of DIPAS changes slightly depending on adsorption configurations as shown in Fig. 2a, b and S3 (ESI†). While one configuration (Fig. S3, ESI†) shows a barrier of 0.02 eV, the other (Fig. 2a and b) shows a barrierless reaction. These slight differences were confirmed by narrowing down the searching region of the transition states. The reaction energy, the energy difference between initial and final states, were calculated based on energy values of each adsorbate on separate slabs. To model favorable reaction paths, we referred to our previous results which utilized both AIMD and CI-NEB calculations to find the decomposition reaction path of DIPAS.^26^

## 3. Results and discussion

### 3.1. Adsorption and decomposition of Si precursors on the WO_3_ surface

The first decomposition reactions of all three Si precursors take place in the same manner, where Si–N breaking is self-catalyzed by the terminal OH groups on the WO_3_ surface as illustrated in Fig. 2. Such a reaction path has been verified in several previous studies of Si ALD reactions on SiO_2_ surfaces.^22–24^ We also reported in our previous study that the Si–N bond of DIPAS breaks over the terminal OH group of WO_3_(001) with an almost barrierless activation energy of 0.02 eV as shown in Fig. S3 (ESI†).^26^ Herein, we used a slightly modified configuration for DIPAS compared to our previous study by rotating the DIPAS molecule on the surface by 180° as shown in Fig. 2a so that all the three precursors adsorb on the surface in the same manner. There is almost no difference in reaction barrier and reaction energy between two configurations, with only slight difference of 0.02 eV in the barrier. As a result of initial adsorption, the Si–N bond cleavages of three precursors were facilitated *via* the same atomic pair interactions on initial adsorption in which N and Si atoms of precursors interact with the H and O atoms of a surface terminal OH group, respectively (Fig. 2). Three precursors show some differences in initial adsorption configurations with respect to the position of alkyl function groups. DIPAS shows the strongest binding energy on the WO_3_ (001) surface as listed in Table 1, which appears to be associated with closer interaction of two alkyl functional groups with the surface due to the molecular conformation of DIPAS as shown in Fig. 2a. On the contrary, BDEAS has the weakest binding energy, which can be also explained by the conformational feature that two alkyl functional groups face up away from the surface. We also examined ligand and molecular volumes as shown in Table S1† to affect the binding energies by steric hindrance. The strongest binding energy of DIPAS is found to be connected to its relatively smaller molecular volume compared to other two precursors. Such a correlation is also found in BDEAS which has the largest molecule size, resulting in the least binding strength on the surface along with the longest bond length of OH and N. The binding energy of the precursor becomes stronger with decrease of the molecular volume, while the effect of ligand size on the binding energies is not seen as clearly as that of molecular one. We note that the larger molecular volume of the precursor increases the steric effect to reduce the initial adsorption. As ALD windows can be estimated based on adsorption energies and reaction energy barriers of precursors,^44^ the upper boundary of ALD window related to precursor deposition is expected to be enlarged in the order of BDEAS, TDMAS, and DIPAS.

**Table 1:** Binding energies Δ*E*_ads_ (eV) and bond lengths *d* (Å) for molecular adsorption of DIPAS, BDEAS, and TDMAS on the hydroxyl-terminated WO_3_ (001) surface. The Δ*E*_ads_ values were calculated based on the reaction equation of precursor (g) + surface → precursor* (where * represents an adsorbed state). The Δ*E*_ads_ + Δ*E*_chem_ values (eV) indicate the sum of precursor binding energies and the reaction energies of dissociative chemisorption of precursors on WO_3_(001). The binding energies of leaving amine molecules on WO_3_(001) are listed in the column of Δ*E*_ads_ (amine) in eV

	Initial adsorption					Δ*E*_ads_ + Δ*E*_chem_	Δ*E*_ads_ (amine)
	Δ*E*_ads_	*d*_Si–N_	*d*_OH–N_	*d*_OH–Si_	*d*_O–H_		
DIPAS	−0.76	1.771	1.666	2.398	1.024	−1.90	−0.89
BDEAS	−0.53	1.772	1.819	2.364	1.002	−3.29	−2.27
TDMAS	−0.61	1.783	1.592	2.347	1.034	−3.18	−2.05

**Fig. 3:**
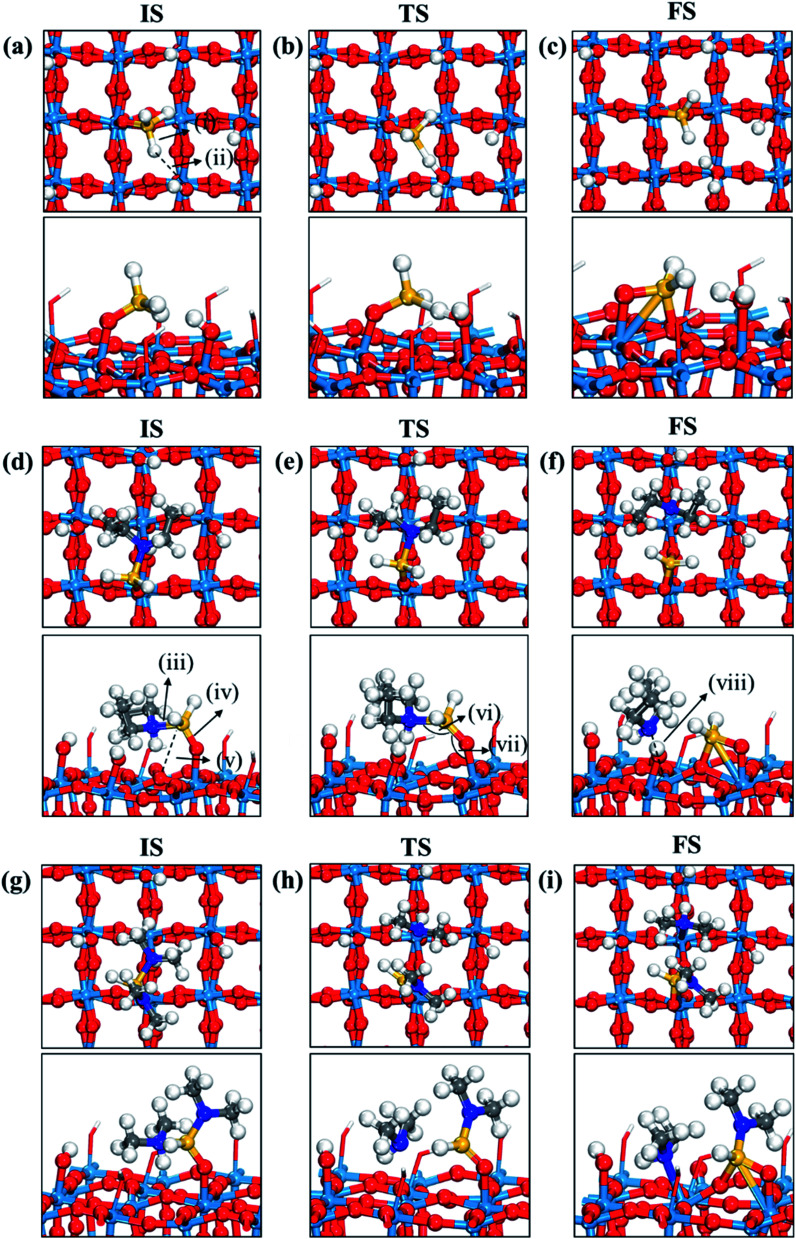
Top and side views of initial (IS), transition (TS), and final (FS) states of the second dissociation step of three precursors such as (a–c) DIPAS, (d–f) BDEAS, (g–i) TDMAS. Terminal hydroxyl groups on the WO_3_ surface are displayed in stick style if there is no direct interaction with adsorbates, in which red and white colors indicate O and H atoms, respectively. The decomposition reaction of DIPAS is redrawn with permission from ref. 26. Red spheres, O; light blue spheres, W; blue spheres, N; yellow spheres, Si; gray spheres, C; white spheres, H. (i) Si–H distance (1.493 (a); 1.856 (b); 1.803 (c)), (ii) H–O distance (3.077 (a); 1.306 (b); 0.983 (c)), (iii) Si–N distance (1.893 (d); 1.987 (e); 3.276 (f); 1.911 (g); 3.160 (h); 3.488 (i)), (iv) terminal O–Si distance (1.589 (d); 1.595 (e); 1.633 (f); 1.595 (g); 1.564 (h); 1.637 (i)), (v) non-terminal O–Si distance (3.457 (d); 3.099 (e); 1.709 (f); 3.394 (g); 3.035 (h); 1.717 (i)), (vi) N–Si–O angle (105.1° (d); 127.8° (e); 101.0° (g); 125.2° (h)), (vii) Si–O–W angle (148.1° (d); 147.3° (e); 103.0° (f); 145.6 (g); 136.8° (h); 114.1° (i)), (viii) N–W distance (2.883 (f); 2.513 (i)). The unit of distance is Å.

**Fig. 4:**
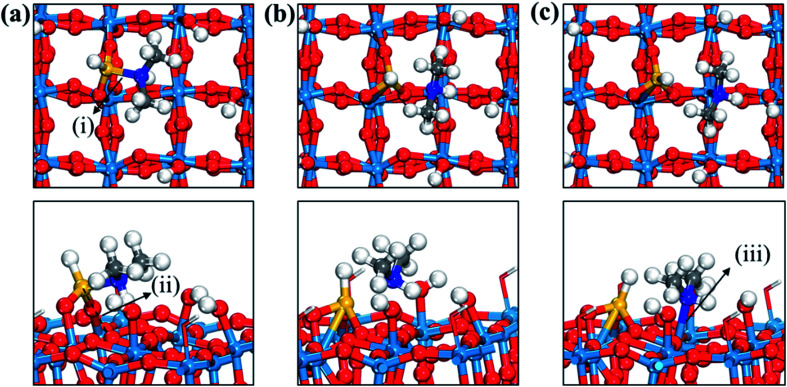
Top and side views of initial (a), transition (b), and final (c) states of the third dissociation step of TDMAS. Terminal hydroxyl groups on the WO_3_ surface are displayed in stick style if there is no direct interaction with adsorbates, in which red and white colors indicate O and H atoms, respectively. Red spheres, O; light blue spheres, W; blue spheres, N; yellow spheres, Si; gray spheres, C; white spheres, H. (i) Si–N distance (1.889 (a); 2.602 (b); 3.572 (c)), (ii) Si–O distance (3.126 (a); 1.731 (b); 1.696 (c)), (iii) N–W distance (3.843 (a); 3.203 (b); 2.442 (c)). The unit of distance is Å.

**Fig. 5:**
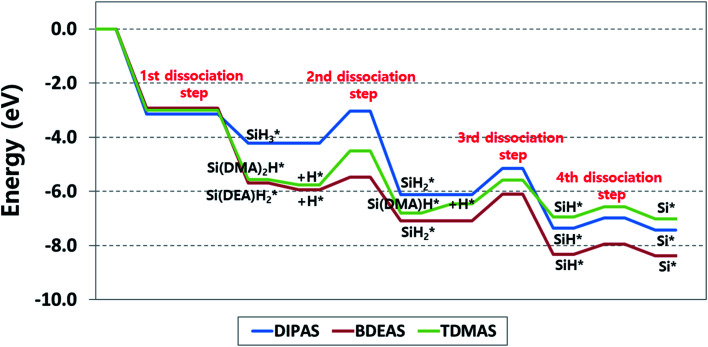
Energy diagram of reaction pathways for decomposition of DIPAS, BDEAS, and TDMAS on the hydroxyl-terminated WO_3_ (001) surface. DMA and DEA indicate dimethylamino and diethylamino ligands, respectively. The reaction energetics of DIPAS from the second to the fourth dissociation steps are redrawn with permission from ref. 26.

Fig. 3 displays the second dissociation step after dissociative chemisorption. The remaining Si fragment of DIPAS, 
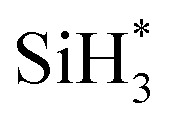
, is decomposed *via* Si–H breaking by a neighbor terminal OH group as we previously reported,^26^ whereas BDEAS and TDMAS still have one or two remaining Si–N bonds to be broken. Previously, Li *et al.* reported that the second Si–N bond scission can occur by an adjacent terminal OH group on the hydroxylated SiO_2_ (001) surface.^24^ However, there is no adjacent terminal OH group available near the Si precursor fragment adsorbed on the WO_3_ (001) surface. The lack of terminal OH groups for the second Si–N breaking step is replenished by surface hydrogen atoms available at the reduced conditions of ALD. A diffusing hydrogen atom on the surface is likely to be attached to the N atom of the amino group, which is an energetically favorable process as represented by +H* in Fig. 5. Once a surface hydrogen atom is added to the N atom, the amino group can be detached from the precursor fragment as a stable amine gas, resulting in bond formation between the Si atom and a surface oxygen atom. From the third dissociation step (Fig. 4), there is no difference between DIPAS and BDEAS as their remaining Si fragments are the same as 
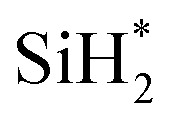
. The Si–H scission of 
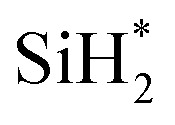
 takes place by reacting with a neighbor surface OH group according to our previous report (Fig. S4a–c, ESI†).^26^ On the other hand, TDMAS has one more Si–N bond which can be cleaved in the same way as the second dissociation step, which is facilitated by taking a hydrogen atom diffusing over the surface. In the fourth dissociation step, all the three precursors reach the same Si fragment, SiH*, which goes through Si–H breaking by a neighbor terminal OH group, leaving a Si seed on the surface as shown in Fig. S4d–f (ESI†).

### 3.2. ALD reaction energetics of Si precursors

To explore the reaction energetics of precursor decomposition with respect to the number of alkylamino ligands, the potential energy surfaces of decomposition of three Si precursors on the OH-covered WO_3_ (001) surface are mapped out in Fig. 5. As we compare three different precursors, all three reaction energetics cannot be clearly depicted in a plot if the molecular energy of a precursor gas or an alternative gas (*i.e.*, SiH_4_) is used as the reference states of all the precursors, because the leaving amine gases following Si–N breaking are different from one another depending on precursor. This causes the energy levels of some intermediates to be inconsistent among precursors in spite of the same reaction. For instance, the Si–H breaking step of SiH* identically appears in the decomposition process of all the precursors, but the energy levels of SiH* become different when silane is used as the reference gas. To resolve such discrepancy, we constructed the potential energy surfaces of all the precursors as shown in Fig. 5 using each corresponding precursor molecule for each reference state along with a hydrogen gas molecule as follows,

1 Precursor (g) + H_2_ (g) + surface → precursor* + 2H*

where * represents an adsorbed state and H_2_ (g) is included to provide H surface species diffusing on the WO_3_ surface to be used for Si–N breaking. We also made a different version of diagram as shown in Fig. S5 (ESI†) by shifting up the entire energy levels of the potential energy surfaces of both BDEAS and TDMAS by the energy differences in the energy level of Si* from DIPAS, as the Si* is the common reaction product that all three precursors have in their respective reaction pathway. As a result, the 
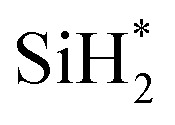
 energies of both BDEAS and DIPAS and the SiH* energies of all the three precursors are fitted to the same energy level, removing the influence of leaving amine gases on each energy level in the potential energy surfaces. The only problem arising from this approach is that the energy levels of initial precursor adsorption on WO_3_(001) are incorrectly adjusted, because this adsorption energy calculation involves the gas molecule energies of each precursor which is set as 0 eV in the *y*-axis without being affected by the energy level shift. Thus, the adsorption strength comparison among precursors has to be made on the straightforward calculation of energy differences between a gas molecule and an adsorbate over the surface, which is shown in Table 1 and Fig. 5.

With the reaction energetics in Fig. 5, we compare reaction energies and barriers across precursors to identify the correlation between the number of amino ligands and reaction energetics. There is no difference in the activation energy of the first step, dissociative chemisorption, where the detachment of the first ligand is barrierless as discussed earlier. However, the reaction energies of BDEAS and TDMAS are thermodynamically more favorable than that of DIPAS, because the Si adsorbates from BDEAS and TDMAS become more stable on the surface by the electron donating effect of alkylamino groups. If the molecular adsorption energies are considered together, the exothermicity trend remains unchanged, which follows the order of BDEAS > TDMAS ≫ DIPAS as tabulated in Table 1. The exothermicity of BDEAS and TDAMS are comparable each other with the small energy difference of 0.1 eV, but DIPAS shows a large energy difference of 1.4–1.5 eV from other two precursors. The main reason is the leaving amine molecules from BDEAS and TDMAS are less bulky than that from DIPAS, leading to much stronger adsorption on the surface as quantified by the binding energies of leaving amine molecules as listed in Table 1. To put it another way, BDEAS and TDMAS can enhance the deposition process, but are likely to be more vulnerable to impurity arising from leaving amine groups as compared to DIPAS, which is was experimentally observed by a previous experimental study to compare mono- and bi-alkylamino silane precursors in the SiN_*x*_ ALD process.^45^

In the second dissociation step, BDEAS shows the lowest activation energy with energy differences of more than 0.7 eV from those of other two precursors (Table 2), suggesting a relative enhancement in the second Si–N breaking kinetics. In the thermodynamic aspect, DIPAS results in the most exothermic reaction with the energy difference of more than 0.8 eV compared to other two precursors. After this reaction step, DIPAS and BDEAS follows the same reaction path, so we can make a final evaluation of these two precursors at this point. The rate determining steps (RDS) are the second dissociation step for DIPAS and the third dissociation step for BDEAS. Overall, both of them are estimated to decompose efficiently as the reaction energetics are favorable both kinetically and thermodynamically. Looking into the details, the initial deposition involving dissociative chemisorption is thermodynamically more favored with BDEAS, whereas the second dissociation step is with DIPAS. In the kinetic aspects, BDEAS is expected to improve the ALD process compared to DIPAS, due to its lower reaction barrier of RDS by 0.2 eV. This suggests that BDEAS is the more potential precursor since it can offer better performance in both the initial deposition process and the reaction energetics of Si seed formation. This difference is in line with a previous experimental observation that the SiH_3_ surface species formed after the first Si–N bond breaking of a mono-alkylamino silane (di-*sec*-butylaminosilane, DSBAS) remains stable up to a relatively high temperature of 400 °C as compared with the temperature required to break the second Si–N bond of di-alkylamino silane (bis(*tert*-butylamino)silane, BTBAS).^46^

**Table 2:** Activation energies *E*_a_ (eV) and reaction energies *E*_rxn_ (eV) of DIPAS, BDEAS, and TDMAS decomposition on the hydroxyl-terminated WO_3_ (001) surface. The values in parenthesis indicate the reaction energies which are calculated using products adsorbed on the separate slabs

	Step 1		Step 2		Step 3		Step 4	
	b *E*_a_	*E*_rxn_	*E*_a_	*E*_rxn_	*E*_a_	*E*_rxn_	*E*_a_	*E*_rxn_
DIPAS^a^	—	−0.47 (−1.14)	1.19	−1.73 (−1.90)	0.98	−0.82 (−1.24)	0.38	0.09 (−0.07)
BDEAS	—	−0.91 (−2.76)	0.47	0.42 (−1.13)	0.98	−0.82 (−1.24)		
TDMAS	—	−0.93 (−2.57)	1.25	−0.06 (−1.05)	0.89	0.09 (−0.48)		

a Based on results in ref. 26.

b The Si–N bond dissociation steps for three Si precursors are calculated to be barrierless reactions.

In the third dissociation step, TDMAS requires one more Si–N bond breaking step, provided that the endothermic H addition step is completed. This is calculated to be slightly more favored kinetically with a reduction of 0.1 eV in the activation energy and less favored thermodynamically by 0.8 eV compared to the third steps of other two precursors. It turns out that TDMAS shows the worst performance among precursors because it results in the ALD reaction with the least overall exothermicity and the highest reaction barrier in RDS which appears in the second dissociation step. If the energy associated with the endothermic H addition step is excluded, the reaction energy of the third dissociation step is 0.02 eV, implying an equal preference for the forward and reverse reactions. The final step, the fourth dissociation step, is the Si–H bond scission of SiH* for all the three precursors. As reported in our previous study of DIPAS, this reaction is slightly endothermic, so both SiH* and Si* are likely to become the final constituent Si seeds of the first monolayer during the SiO_2_ ALD process on tungsten oxide surfaces.^26^ In the following ALD cycle, these Si seeds are converted into a SiO_2_ layer by oxidizing agents such as O_3_ or H_2_O.

### 3.3. Comparison of WO_3_ and SiO_2_ surfaces in SiO_2_ ALD reactions

To investigate the surface dependent behavior of Si ALD processes, we compare the ALD reaction energetics of BDEAS and TDMAS on the WO_3_ (001) surface with that on the SiO_2_ surfaces. To the best of our knowledge, the full reaction energetics of BDEAS decomposition on SiO_2_ surfaces have not been reported to date. Instead, the first Si–N dissociation step of BDEAS on the OH-covered Si (001) surface was computationally studied by S. B. Baek *et al.* where the reaction barrier and reaction energy are 0.52 and −0.95 eV, respectively.^22^ The reaction energies are similar between the OH-covered Si and WO_3_ surfaces, with a small energy difference of 0.04 eV. However, there is a distinctive difference in energy barrier between two surfaces, given that the first Si–N bond scission of BDEAS on the OH-covered WO_3_ (001) surface is a barrierless reaction. The full reaction energetics of TDMAS on the hydroxylated SiO_2_ (001) surface were previously reported,^24^ which is compared with our results in Fig. S6 (ESI†). We found that the OH-covered WO_3_ surface shows a quite distinctive behavior from the OH-covered SiO_2_ surface due to electronic and structural differences. While the WO_3_ surface easily breaks the first Si–N bond of TDMAS with a barrierless reaction, the SiO_2_ surface requires a high activation energy of 0.83 eV for the first Si–N bond cleavage along with an endothermic reaction energy of 0.43 eV. This is likely to come from a different electronic effect between two surfaces, given that there is not much difference in adsorption configuration between two surfaces. In contrast, the SiO_2_ surface decomposes the Si–N bond more efficiently than the WO_3_ surface in the second dissociation step. The activation and reaction energy of Si–N breaking on the OH-covered SiO_2_ surface are 0.83 eV and 0.35 eV lower than that on the OH-covered WO_3_ surface, respectively. This discrepancy results from the geometrical difference in the surface OH group arrangement between two surfaces, as the terminal OH groups are distributed more densely near the reaction site on the SiO_2_ surface than on the WO_3_ surface. Lastly, there is a clear difference in the third dissociation step where the SiO_2_ surface shows a large endothermic reaction with a significantly high reaction barrier of 4.05 eV. On the other hand, the reaction is slightly endothermic with a moderate reaction barrier of 0.9 eV on the WO_3_ surface. Thus, TDMAS can be one of potential precursors on the WO_3_ surface, but not on the SiO_2_ surface according to DFT calculation results.

## 4. Conclusion

In this work, the ALD reactions of three different alkylamino silane precursors such as DIPAS, BDEAS, and TDMAS have been exploited on the hydroxyl-terminated WO_3_ (001) surface using DFT methods. Specifically, we probed the influence of the different number of alkylamino ligands, ranging from mono- to tri-alkylamino substituted precursors, on the reaction energetics of the SiO_2_ ALD process. The molecular adsorption strength of precursors on WO_3_ (001) associated with the ALD deposition rate is found to decrease in the order of DIPAS, TDMAS, and BDEAS. However, as the hydroxyl-terminated tungsten oxide surface induces a barrierless dissociative chemisorption for all the precursors, the effective adsorption strength can be represented by the sum of the molecular adsorption energy of precursors and the reaction energy of the first Si–N breaking step. According to the effective adsorption strength, BDEAS and TDMAS are bound more strongly to the surface than DIPAS, so ALD deposition rate is estimated to be enhanced by using BDEAS and TDMAS. However, we found the stronger binding strengths of BDEAS and TDMAS to arise from the stronger adsorption of leaving amine molecules, implying that BDEAS and TDMAS possibly result in more impurity than DIPAS. In the second dissociation step, DIPAS undergoes the Si–H bond cleavage of SiH_3_ adsorbate with a high reaction barrier of 1.19 eV which turns out to be the RDS of the DIPAS ALD reactions, whereas BDEAS still has one more Si–N bond which can be broken with a relatively low reaction barrier of 0.47 eV. As a result, the RDS of the BDEAS ALD reactions appears in the third dissociation step, the Si–H bond cleavage of SiH_2_ adsorbate, whose reaction barrier is lower than that of the RDS of DIPAS by 0.2 eV, suggesting that BDEAS is kinetically more effective than DIPAS. The RDS of TDMAS was identified to be the second dissociation step, a Si–N bond breaking step, in contrast to other two precursors which have the RDS in the Si–H bond breaking step. The activation energy of the TDMAS RDS is calculated to be the highest among three precursors. Moreover, the reaction pathways of TDMAS decomposition contain an additional endothermic reaction in the third dissociation step. Thus, the ALD performance of TDMAS is predicted to be relatively less efficient than those of other two precursors. Our energetic analysis of three precursors suggest that bi-alkylamino substitution is a potential starting structure to develop a new Si precursor, provided that one finds an optimal point to maximize the adsorption strength of precursor molecule and minimize that of leaving amine molecule.

## Conflicts of interest

There are no conflicts of interest to declare.

## Supplementary Material

RA-010-D0RA01635G-s001
