# Supplementary material for: First-principles study of the surface reactions of aminosilane precursors over WO3(001) during atomic layer deposition of SiO2
Source: RSC Adv. 2020 Apr 27;10(28):16584–92. doi: 10.1039/d0ra01635g (PMC9122564; doi:10.1039/d0ra01635g)
Supplement: RA-010-D0RA01635G-s001 [file RA-010-D0RA01635G-s001.pdf]

Electronic Supplementary Material (ESI) for RSC Advances.
This journal is © The Royal Society of Chemistry 2020

First-principles study of the surface reactions of aminosilane precursors over $WO_3(001)$ during atomic layer deposition of SiO_2

Kyungtae Lee[a], Youngseon Shim[a*]

[a]CSE Team, Data & Information Technology (DIT) Center, Samsung Electronics Co. Ltd., 130 Samsung-ro, Suwon, Gyeonggi-do, 16678, South Korea. E-mail: ys1231.shim@samsung.com

Table S1. Volumes of precursor molecules and the corresponding amino ligand, which were measured at the medium level of grid resolution using the Connolly surface task in Materials Studio software.

	molecular volume (Å^3)	amino ligand volume (Å^3)
DIPAS	169.34	130.88
BDEAS	215.30	92.80
TDMAS	191.04	56.26

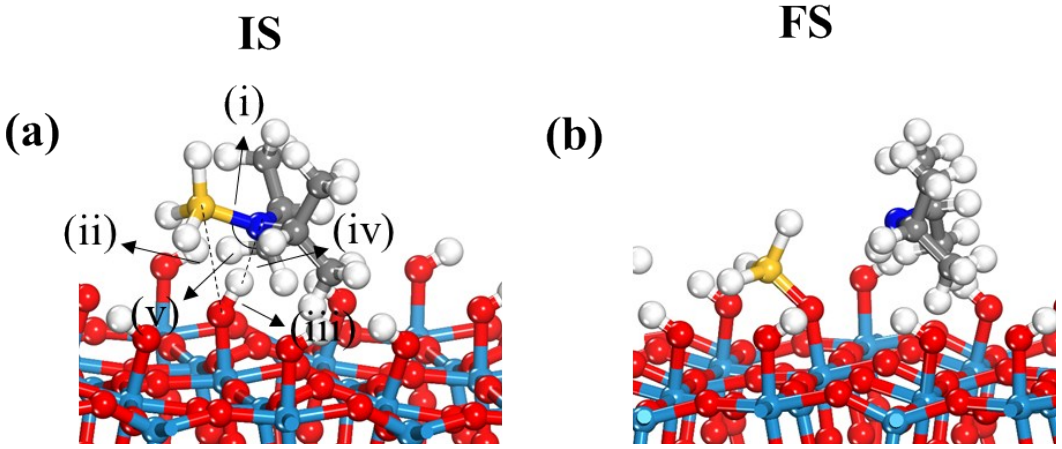

Fig. S1. Initial (IS) and final (FS) states of the first dissociation step, dissociative chemisorption, of DIPAS using the force convergence criterion of 0.03 eV Å^{-1}. Red spheres, O; light blue spheres, W; blue spheres, N; yellow spheres, Si; gray spheres, C; white spheres, H. (i) Si-N distance (1.772, a; 4.077, b), (ii) Si-O distance (3.098, a; 1.648, b), (iii) O-H distance (1.026, a; 2.681, b), (iv) N-H distance (1.663, a; 1.023, b), and (v) H-N-Si angle (89.5°, a). The unit of distance is Å.

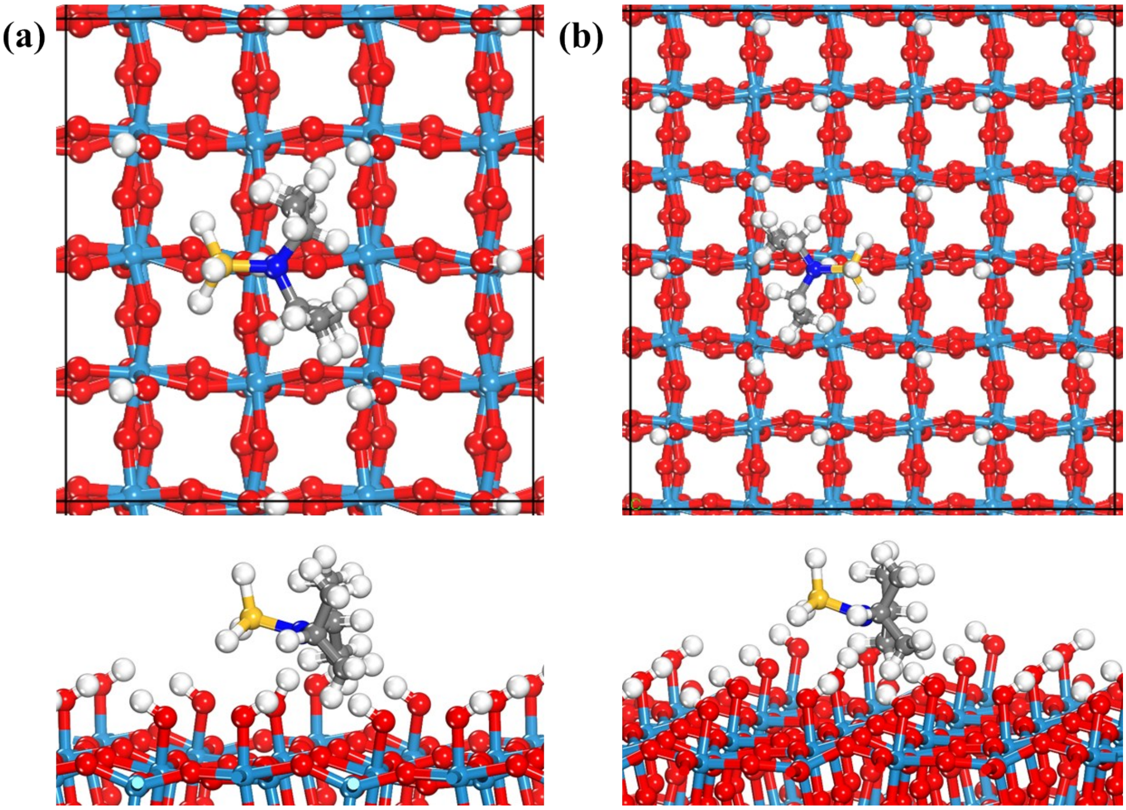

Fig. S2. Adsorption of DIPAS on the (a) 4 × 4 and (b) 6 × 6 cell units of WO_3 (001) surface, respectively, where the black line indicates the cell boundaries of surface slabs. Red spheres, O; light blue spheres, W; blue spheres, N; yellow spheres, Si; gray spheres, C; white spheres, H.

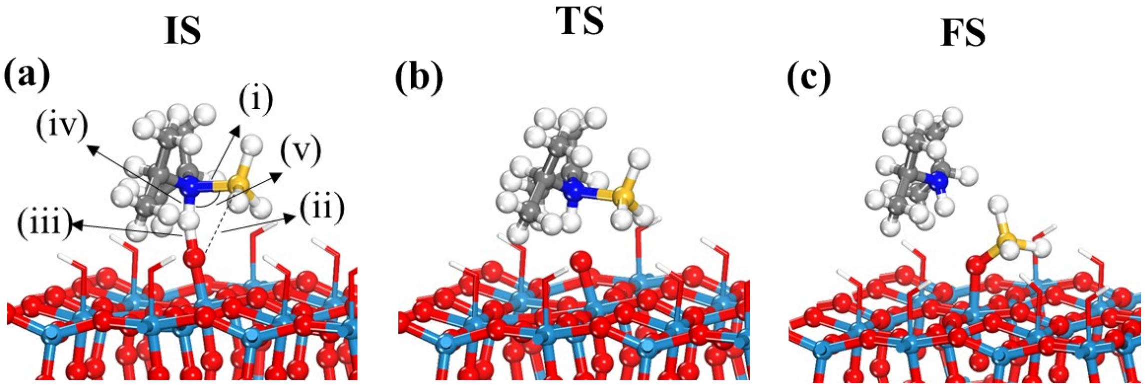

Fig. S3. Initial (IS), transition (TS), final (FS) states of the first dissociation step, dissociative chemisorption, of DIPAS, which was reported in ref 26. Terminal hydroxyl groups on the WO_3 surface are displayed in stick style if there is no direct interaction with adsorbates, in which red and white colors indicate O and H atoms, respectively. The decomposition reaction of DIPAS is redrawn with permission from ref 26. Red spheres, O; light blue spheres, W; blue spheres, N; yellow spheres, Si; gray spheres, C; white spheres, H. (i) Si-N distance (1.826, a; 1.847, b; 3.627, c), (ii) Si-O distance (3.099, a; 2.854, b; 1.653, c), (iii) O-H distance (1.342, a; 1.455, b; 2.579, c), (iv) N-H distance (1.181, a; 1.122, b; 1.021, c), and (v) H-N-Si angle (95.8°, a). The unit of distance is Å.

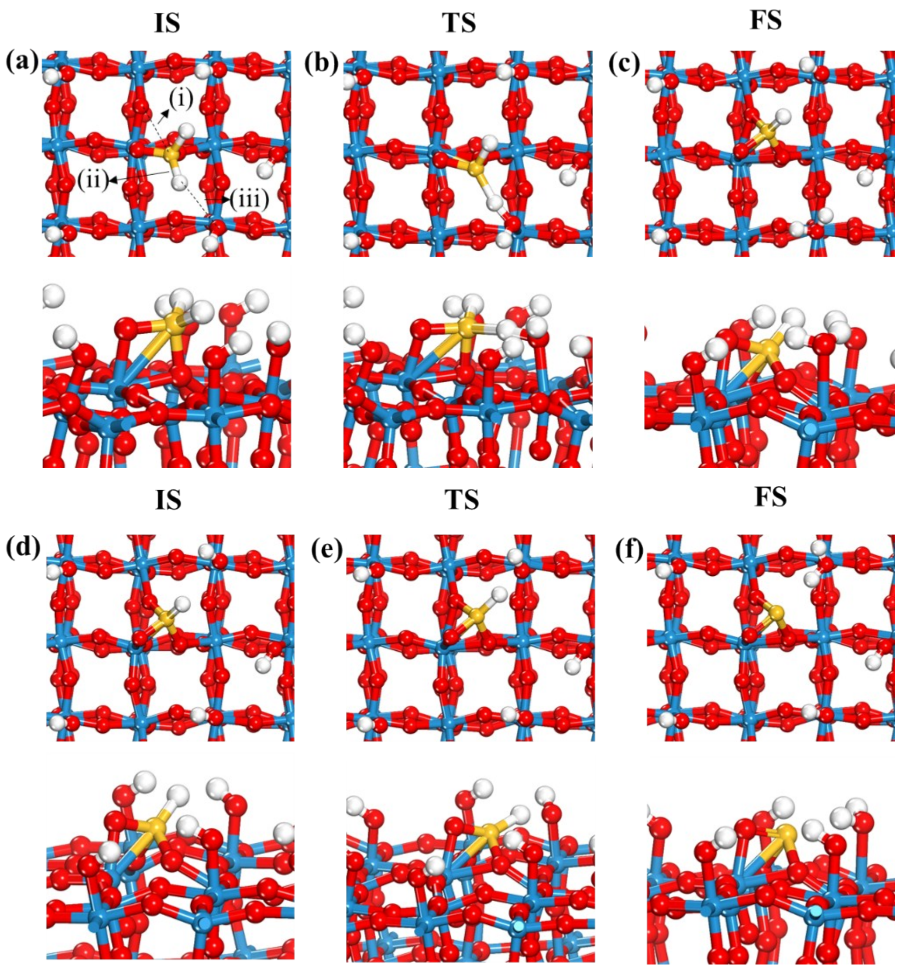

Fig. S4. Top and side views of initial (IS), transition (TS), and final (FS) states of (a-c) SiH_2 and (d-f) SiH dissociation. These reactions are redrawn with permission from ref 26. Red spheres, O; light blue spheres, W; blue spheres, N; yellow spheres, Si; gray spheres, C; white spheres, H. (i) Si-O distance (3.371, a; 3.596, b; 1.676, c), (ii) Si-H distance (1.483, a; 1.831, b; 5.052, c; 1.466, d; 1.618, e; 2.426, f), and (iii) H-O distance (2.933, a; 1.279, b; 0.975, c; 2.529, d; 1.591, e; 1.006, f). The unit of distance is Å.

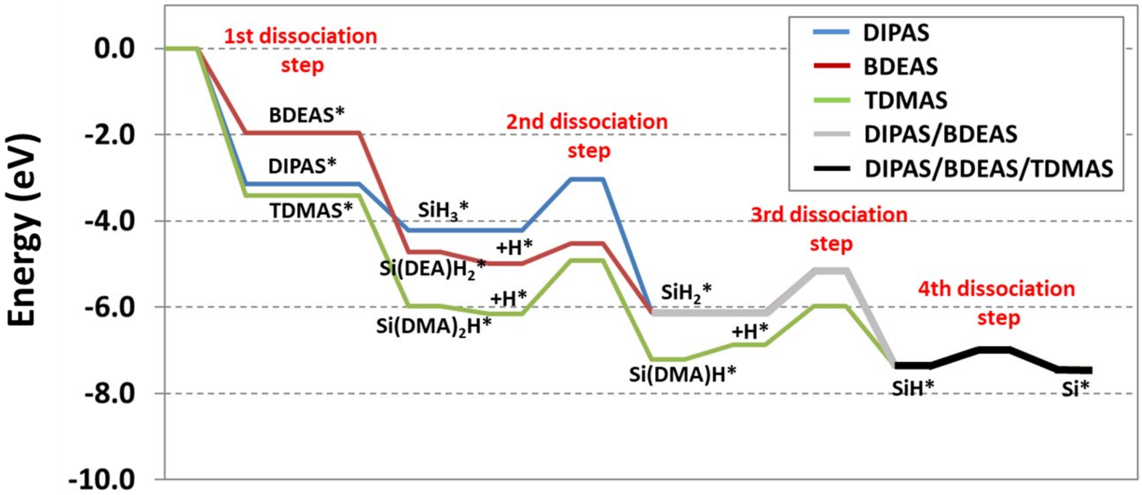

Fig. S5. Energy diagram of redrawn reaction pathways of Fig. 5 by adjusting the energy levels of Si*.

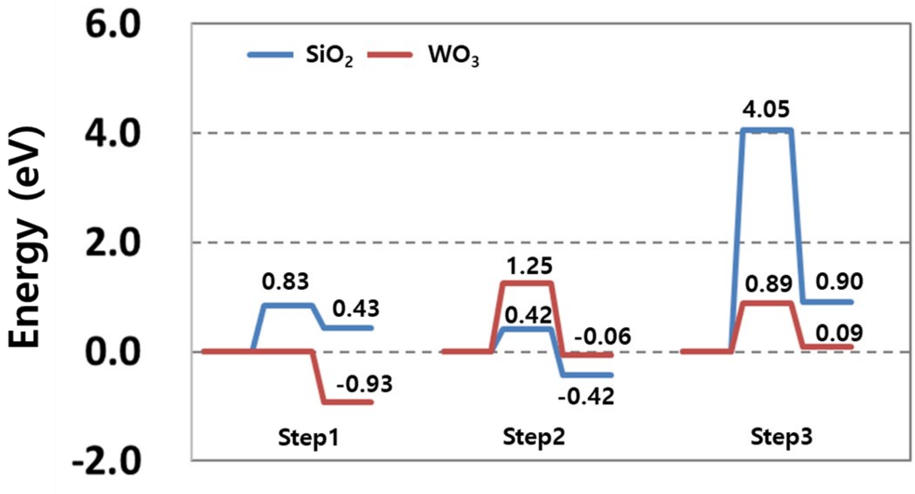

Fig. S6. Comparison of the reaction energetics of three sequential Si-N dissociation steps of TDMAS between on the hydroxyl-terminated WO_3 (001) surface and on the hydroxyl-terminated SiO_2 (001) surface. The reaction barriers and reaction energies are expressed on the energy diagrams in eV. The SiO_2 results are based on ref 24.

Supplementary Note: Coordinates of the crystal structure, surface slab, and precursor molecules

(1) The bulk structure of WO_3

lattice constant

7.3000001906999996	0.0000000000000000	0.0000000000000000
0.0000000000000000	7.5300002097999998	0.0000000000000000
-0.1206323988000000	0.0000000000000000	7.6790523626000002

fractional coordinates

O	0.293063909	0.039349102	0.007531346
O	0.706582443	0.516557281	0.006202568
O	-0.000878972	0.043711820	0.208394873
O	-0.000330407	0.455971636	0.210437265
O	0.701004754	0.240381228	0.232793485
O	0.210031489	0.760018097	0.213498455
O	0.289968496	0.260018068	0.286501530
O	0.798995246	0.740381214	0.267206500
O	0.500878972	0.543711796	0.291605127
O	0.500330407	0.955971666	0.289562735
O	0.793417557	0.016557291	0.493797430
O	0.206936091	0.539349124	0.492468652
O	0.793063879	0.460650905	0.507531318
O	0.206582413	-0.016557281	0.506202540
O	0.499669593	0.044028358	0.710437295
O	0.499121028	0.456288174	0.708394903
O	0.201004768	0.259618757	0.732793500
O	0.710031475	0.739981903	0.713498470
O	0.789968525	0.239981917	0.786501530
O	0.298995217	0.759618786	0.767206500
O	0.000330407	0.544028334	0.789562705
O	0.000878972	0.956288204	0.791605097
O	0.293417587	0.483442719	0.993797460
O	0.706936121	0.960650876	0.992468682
W	0.248972750	0.523359782	0.217836115
W	0.751701312	0.971607136	0.220673525
W	0.748298688	0.471607136	0.279326460
W	0.251027250	0.023359760	0.282163885
W	0.748972780	0.976640218	0.717836085
W	0.251701282	0.528392864	0.720673510
W	0.248298703	0.028392872	0.779326490
W	0.751027220	0.476640248	0.782163915

(2) The hydroxylated-terminated WO_3 (001) surface slab

lattice constant

14.6000003800000009	0.0000000000000000	0.0000000000000000
0.0000000000000000	15.0600004199999997	0.0000000000000000
-0.5497570404222200	0.0000000000000000	34.9956821221776835

fractional coordinates

O	0.140538000	0.020000000	0.020870300
O	0.640538000	0.020000000	0.020870300
O	0.860538000	0.255000000	0.020870300
O	0.360538000	0.255000000	0.020870300

O	0.640538000	0.520000000	0.020870300
O	0.140538000	0.520000000	0.020870300
O	0.360538000	0.755000000	0.020870300
O	0.860538000	0.755000000	0.020870300
O	0.500538000	0.012500000	0.062561800
O	0.000538000	0.012500000	0.062561800
O	0.000538000	0.237500000	0.062561800
O	0.500538000	0.237500000	0.062561800
O	0.000538000	0.512500000	0.062561800
O	0.500538000	0.512500000	0.062561800
O	0.000538000	0.737500000	0.062561800
O	0.500538000	0.737500000	0.062561800
O	0.355538000	0.105000000	0.065853200
O	0.855538000	0.105000000	0.065853200
O	0.105538000	0.395000000	0.065853200
O	0.605538000	0.395000000	0.065853200
O	0.355538000	0.605000000	0.065853200
O	0.855538000	0.605000000	0.065853200
O	0.105538000	0.895000000	0.065853200
O	0.605538000	0.895000000	0.065853200
O	0.145538000	0.145000000	0.072436000
O	0.645538000	0.145000000	0.072436000
O	0.395538000	0.355000000	0.072436000
O	0.895538000	0.355000000	0.072436000
O	0.145538000	0.645000000	0.072436000
O	0.645538000	0.645000000	0.072436000
O	0.395538000	0.855000000	0.072436000
O	0.895538000	0.855000000	0.072436000
O	0.750538000	0.262500000	0.075727500
O	0.250538000	0.262500000	0.075727500
O	0.750538000	0.487500000	0.075727500
O	0.250538000	0.487500000	0.075727500
O	0.250538000	0.762500000	0.075727500
O	0.750538000	0.762500000	0.075727500
O	0.250538000	0.987500000	0.075727500
O	0.750538000	0.987500000	0.075727500
O	0.390538000	0.005000000	0.117418900
O	0.890538000	0.005000000	0.117418900
O	0.610538000	0.270000000	0.117418900
O	0.110538000	0.270000000	0.117418900
O	0.390538000	0.505000000	0.117418900
O	0.890538000	0.505000000	0.117418900
O	0.110538000	0.770000000	0.117418900
O	0.610538000	0.770000000	0.117418900
O	0.390538000	0.230000000	0.130584600
O	0.890538000	0.230000000	0.130584600
O	0.610538000	0.495000000	0.130584600
O	0.110538000	0.495000000	0.130584600
O	0.390538000	0.730000000	0.130584600
O	0.890538000	0.730000000	0.130584600
O	0.610538000	0.995000000	0.130584600
O	0.110538000	0.995000000	0.130584600
O	0.250538000	0.012500000	0.172276100
O	0.750538000	0.012500000	0.172276100
O	0.250538000	0.237500000	0.172276100
O	0.750538000	0.237500000	0.172276100
O	0.750538000	0.512500000	0.172276100
O	0.250538000	0.512500000	0.172276100
O	0.750538000	0.737500000	0.172276100

O	0.250538000	0.737500000	0.172276100
O	0.605538000	0.145000000	0.175567500
O	0.105538000	0.145000000	0.175567500
O	0.355538000	0.355000000	0.175567500
O	0.855538000	0.355000000	0.175567500
O	0.105538000	0.645000000	0.175567500
O	0.605538000	0.645000000	0.175567500
O	0.855538000	0.855000000	0.175567500
O	0.355538000	0.855000000	0.175567500
O	0.395538000	0.105000000	0.182150300
O	0.895538000	0.105000000	0.182150300
O	0.645538000	0.395000000	0.182150300
O	0.145538000	0.395000000	0.182150300
O	0.395538000	0.605000000	0.182150300
O	0.895538000	0.605000000	0.182150300
O	0.645538000	0.895000000	0.182150300
O	0.145538000	0.895000000	0.182150300
O	0.500538000	0.262500000	0.185441800
O	0.000538000	0.262500000	0.185441800
O	0.500538000	0.487500000	0.185441800
O	0.000538000	0.487500000	0.185441800
O	0.000538000	0.762500000	0.185441800
O	0.500538000	0.762500000	0.185441800
O	0.500538000	0.987500000	0.185441800
O	0.000538000	0.987500000	0.185441800
O	0.146327647	0.253190987	0.237957448
O	0.646180283	0.252355615	0.237944125
O	0.859851524	0.482523717	0.237649369
O	0.359633533	0.482180574	0.237649271
O	0.646298690	0.752875545	0.237949108
O	0.146142960	0.752665203	0.237956281
O	0.360002626	0.982609879	0.237666887
O	0.859952137	0.982424309	0.237670984
O	0.143982194	0.027251879	0.238945223
O	0.643722075	0.027307895	0.238988401
O	0.357063675	0.240843820	0.239549943
O	0.856777971	0.241252918	0.239461383
O	0.143702856	0.527069103	0.239008258
O	0.643661910	0.527664758	0.238931926
O	0.356731047	0.741203366	0.239449674
O	0.857193020	0.740904506	0.239538602
O	0.499826635	0.025925857	0.287530240
O	-0.000212598	0.025904591	0.287248573
O	0.503948369	0.226122571	0.285583740
O	0.003966358	0.226384649	0.285448003
O	0.499583670	0.525726982	0.287250411
O	-0.000313294	0.525800620	0.287510131
O	0.004004941	0.726181881	0.285623383
O	0.503954411	0.726378750	0.285440897
O	0.352765669	0.118393327	0.295291065
O	0.852793486	0.118617685	0.295002921
O	0.104276146	0.383302572	0.286814823
O	0.604247372	0.383095509	0.286337067
O	0.852706806	0.618550312	0.295190107
O	0.352690782	0.618577874	0.294916665
O	0.104283452	0.883117634	0.286602440
O	0.604394189	0.883318407	0.286613676
O	0.150661073	0.131814159	0.300506927
O	0.650939414	0.131822274	0.300603855

O	0.403101321	0.369073354	0.295755821
O	0.903205205	0.369246536	0.295669546
O	0.650367660	0.631797885	0.300659932
O	0.150900038	0.631853709	0.300484595
O	0.903263429	0.869097518	0.295744333
O	0.403475311	0.869054713	0.295589882
O	0.255739862	0.274370766	0.302179971
O	0.755695492	0.274686859	0.302244953
O	0.750191773	0.476713461	0.300546831
O	0.250367455	0.476783837	0.300823042
O	0.755726984	0.774405949	0.302112784
O	0.255718152	0.774713788	0.302279831
O	0.250421254	0.976564772	0.300548625
O	0.750504601	0.976766670	0.300726824
O	0.888841964	0.999235729	0.356031759
O	0.387316237	0.997271276	0.356340173
O	0.111321589	0.268780365	0.349437387
O	0.610103829	0.267909178	0.349373401
O	0.886980174	0.497699540	0.356353155
O	0.388801059	0.499365359	0.356203963
O	0.611655547	0.769018276	0.349411869
O	0.110112315	0.767469202	0.349505712
O	0.407859215	0.240409467	0.348525319
O	0.907953727	0.240217874	0.348310148
O	0.605177192	0.487577841	0.346657336
O	0.105434132	0.489230751	0.347078772
O	0.907818382	0.739965521	0.348564590
O	0.407863073	0.739922794	0.348289444
O	0.105216324	0.987993069	0.346698116
O	0.605531724	0.988530915	0.346972365
O	0.245877372	0.021425932	0.409505949
O	0.746831892	0.021841490	0.408835004
O	0.756516700	0.231962512	0.395568493
O	0.257280185	0.232945902	0.397294786
O	0.246873341	0.521665681	0.409025930
O	0.745657549	0.521487732	0.409835732
O	0.256533225	0.731858343	0.395663698
O	0.757435055	0.732805829	0.397539342
O	0.104785474	0.128452116	0.395909792
O	0.604453492	0.127758800	0.397286585
O	0.857550707	0.368187571	0.408205746
O	0.360084392	0.369100607	0.407234234
O	0.104551512	0.627817528	0.397781036
O	0.604861445	0.628446042	0.395731155
O	0.357510603	0.868078912	0.408528636
O	0.860185565	0.869037786	0.407136292
O	0.396983311	0.126556241	0.410929391
O	0.896680156	0.127868087	0.411828801
O	0.649227868	0.382564149	0.407629674
O	0.150773746	0.382935912	0.408329289
O	0.896909836	0.626649849	0.411138860
O	0.396838179	0.627990874	0.411989145
O	0.651274648	0.882942895	0.408363537
O	0.149214629	0.882748655	0.407500871
O	0.503420627	0.271964068	0.416901441
O	0.003305380	0.273430899	0.415725559
O	0.008397858	0.482202438	0.417771986
O	0.509474880	0.484696052	0.418549182
O	0.003616366	0.771909404	0.417038426

O	0.503414580	0.773580690	0.415468769
O	0.508579763	0.982253736	0.417847597
O	0.009273393	0.984638140	0.418507562
O	0.152636397	0.233077626	0.455480919
O	0.657660386	0.235835373	0.455386854
O	0.372388097	0.494904071	0.463384816
O	0.873079336	0.496220204	0.463531170
O	0.158037806	0.737005679	0.455590459
O	0.651647865	0.732885785	0.455466949
O	0.372908425	0.997093098	0.463481739
O	0.871939538	0.994881241	0.463208221
W	0.124538000	0.263000000	0.061464600
W	0.624538000	0.263000000	0.061464600
W	0.624538000	0.763000000	0.061464600
W	0.124538000	0.763000000	0.061464600
W	0.377038000	0.483500000	0.062342300
W	0.877038000	0.483500000	0.062342300
W	0.877038000	0.983500000	0.062342300
W	0.377038000	0.983500000	0.062342300
W	0.374038000	0.233500000	0.075946900
W	0.874038000	0.233500000	0.075946900
W	0.874038000	0.733500000	0.075946900
W	0.374038000	0.733500000	0.075946900
W	0.626538000	0.013000000	0.076824600
W	0.126538000	0.013000000	0.076824600
W	0.126538000	0.513000000	0.076824600
W	0.626538000	0.513000000	0.076824600
W	0.874538000	0.487000000	0.171178900
W	0.374538000	0.487000000	0.171178900
W	0.374538000	0.987000000	0.171178900
W	0.874538000	0.987000000	0.171178900
W	0.627038000	0.266500000	0.172056600
W	0.127038000	0.266500000	0.172056600
W	0.127038000	0.766500000	0.172056600
W	0.627038000	0.766500000	0.172056600
W	0.624038000	0.016500000	0.185661200
W	0.124038000	0.016500000	0.185661200
W	0.124038000	0.516500000	0.185661200
W	0.624038000	0.516500000	0.185661200
W	0.376538000	0.237000000	0.186538900
W	0.876538000	0.237000000	0.186538900
W	0.876538000	0.737000000	0.186538900
W	0.376538000	0.737000000	0.186538900
W	0.128969591	0.264900573	0.288202142
W	0.628973909	0.264909405	0.288177415
W	0.129004106	0.764816170	0.288213169
W	0.628936560	0.764968974	0.288180365
W	0.875814259	0.485698369	0.287941192
W	0.375851127	0.485509313	0.287945558
W	0.876075212	0.985640666	0.287980308
W	0.376038694	0.985560441	0.287968427
W	0.877012071	0.236444329	0.292452336
W	0.377084083	0.236212745	0.292573620
W	0.877142772	0.736418618	0.292552233
W	0.376992180	0.736372092	0.292445759
W	0.128220949	0.015585898	0.291778433
W	0.628319262	0.015701992	0.291800924
W	0.628018402	0.515653179	0.291743503
W	0.128145048	0.515635650	0.291808607

W	0.871898227	0.492818972	0.407749156
W	0.372650373	0.493513275	0.407557867
W	0.872699261	0.993641451	0.407423098
W	0.372052139	0.992537580	0.407742269
W	0.130926252	0.263166330	0.401469605
W	0.630845256	0.262763733	0.401373375
W	0.130989901	0.762776650	0.401505536
W	0.631226945	0.763310909	0.401433747
W	0.119493226	0.009299508	0.396359210
W	0.619572125	0.008627512	0.396662452
W	0.119517657	0.508681392	0.396799543
W	0.619513825	0.509285910	0.396301107
W	0.884888300	0.243846555	0.397804193
W	0.385763853	0.243336576	0.398103462
W	0.385026552	0.743725348	0.397810039
W	0.885979111	0.743441391	0.398149029
H	0.100685082	0.233794707	0.472547509
H	0.110223944	0.748007557	0.474041349
H	0.428639885	0.001211883	0.478847582
H	0.428713717	0.491796244	0.478424184
H	0.609969788	0.247217056	0.473865465
H	0.599228498	0.731150913	0.472229812
H	0.928072646	0.991680767	0.478409416
H	0.929205095	0.498291886	0.478718270

(3) DIPAS

lattice constant

20.0000000000000000	0.0000000000000000	0.0000000000000000
0.0000000000000000	20.0000000000000000	0.0000000000000000
0.0000000000000000	0.0000000000000000	20.0000000000000000

fractional coordinates

H	0.476434550	0.430445950	0.408550150
H	0.564075000	0.510727600	0.388705450
H	0.446632200	0.545388500	0.390263850
H	0.606837350	0.609499550	0.541214050
H	0.401735150	0.446890650	0.551100950
H	0.568563650	0.583617550	0.617411700
H	0.369437750	0.501309300	0.488404700
H	0.649853600	0.554704600	0.596969850
H	0.348863550	0.515202800	0.573539600
H	0.467366200	0.540406650	0.599882100
H	0.606219350	0.493213850	0.499022800
H	0.415504450	0.625234800	0.482954100
H	0.531746250	0.455067600	0.628073150
H	0.392318900	0.634515350	0.568404500
H	0.615177800	0.433322950	0.607255950
H	0.547534150	0.401519650	0.558652650
H	0.476270550	0.650559950	0.542762650
C	0.600623400	0.567200500	0.575768150
C	0.389309550	0.499228400	0.539780550
C	0.570898500	0.506514150	0.539384100
C	0.450745700	0.544536150	0.547438550
C	0.565919500	0.445574950	0.585941150
C	0.432697450	0.618060300	0.534750450
N	0.507341000	0.522375300	0.505487100
Si	0.497894400	0.501262850	0.422128200

(4) BDEAS

lattice constant

20.0000000000000000	0.0000000000000000	0.0000000000000000
0.0000000000000000	20.0000000000000000	0.0000000000000000
0.0000000000000000	0.0000000000000000	20.0000000000000000

fractional coordinates

C	0.399774976	0.520516595	0.530685742
C	0.438419280	0.585165573	0.544477678
C	0.431135928	0.495959155	0.411879365
C	0.363766779	0.518324922	0.383959466
C	0.621130241	0.432879672	0.566325801
C	0.662419248	0.377501204	0.533092869
C	0.596814090	0.501509691	0.465399203
C	0.650405108	0.553425426	0.481600244
H	0.475906956	0.395270478	0.577212273
H	0.499719249	0.369103230	0.458818821
H	0.393742605	0.492499671	0.577838186
H	0.348599266	0.532268685	0.513795562
H	0.446817433	0.613854980	0.498243833
H	0.487515841	0.573726586	0.566454365
H	0.410602287	0.617393910	0.579373395
H	0.449647082	0.452950317	0.382627181
H	0.467106323	0.537180392	0.402527880
H	0.325420686	0.479133383	0.388094167
H	0.370030074	0.531136993	0.330813936
H	0.345286098	0.563526307	0.409244161
H	0.594847394	0.412442148	0.610317445
H	0.654918613	0.471923018	0.585874467
H	0.687156067	0.395684119	0.487400507
H	0.630579171	0.334932361	0.518933850
H	0.701639352	0.359095180	0.567027711
H	0.554120093	0.527955597	0.443033965
H	0.616202839	0.467688243	0.425798301
H	0.662925662	0.581218191	0.435784567
H	0.696909275	0.530565073	0.499964909
H	0.632750136	0.589409006	0.519288241
N	0.570879437	0.464099078	0.523127295
N	0.431110067	0.475167934	0.482295147
Si	0.494515393	0.423389034	0.510531016

(5) TDMAS

lattice constant

20.0000000000000000	0.0000000000000000	0.0000000000000000
0.0000000000000000	20.0000000000000000	0.0000000000000000
0.0000000000000000	0.0000000000000000	20.0000000000000000

fractional coordinates

H	0.488307451	0.505049546	0.444006210
H	0.616204012	0.417419904	0.521892985
H	0.569449651	0.590643231	0.429958884
H	0.386598927	0.558466438	0.462689588
H	0.594870071	0.365306110	0.591277303
H	0.567752567	0.343444942	0.509230389
H	0.593107223	0.646048206	0.495911256
H	0.377097404	0.623084403	0.523563618
H	0.652971493	0.589751927	0.461166876

H	0.331279786	0.546981182	0.531734481
H	0.473860277	0.364445559	0.629200712
H	0.614733966	0.594398549	0.610890736
H	0.430969386	0.604741695	0.637675859
H	0.449401532	0.342496096	0.546333652
H	0.673831604	0.538769674	0.573990080
H	0.383466845	0.529806652	0.646750943
H	0.472354505	0.526872495	0.651743676
H	0.604082939	0.506023440	0.618942145
H	0.415935635	0.415895684	0.584661101
C	0.576488608	0.386203489	0.543413825
C	0.599798087	0.594851119	0.475634919
C	0.380123337	0.568195892	0.516402250
C	0.461232939	0.385316454	0.579501766
C	0.430231330	0.550663196	0.625554106
C	0.619945854	0.545861926	0.584543530
N	0.516002889	0.425317414	0.553405557
N	0.434678400	0.538882677	0.554089004
N	0.580942896	0.543474377	0.523351592
Si	0.504412472	0.503151146	0.517166527
